# Assessment of the relationship between serum uric acid levels and liver enzymes activity in Bangladeshi adults

**DOI:** 10.1038/s41598-021-99623-z

**Published:** 2021-10-11

**Authors:** Noyan Hossain Molla, Rahanuma Raihanu Kathak, Abu Hasan Sumon, Zitu Barman, Ananya Dutta Mou, Akibul Hasan, Firoz Mahmud, Khandaker Atkia Fariha, Nurshad Ali

**Affiliations:** Department of Biochemistry and Molecular Biology, Shahjalal University of Science and Technology, Sylhet, 3114 Bangladesh

**Keywords:** Medical research, Epidemiology

## Abstract

Serum uric acid (SUA) level has been suggested to be associated with cardiovascular disease, diabetes and metabolic syndrome. However, little is known about the relationship between SUA and liver enzymes activity in the general population. The present study aimed to assess the relationship between SUA and serum liver enzymes in an adult population in Bangladesh. In this cross-sectional study, a total of 410 blood samples were collected from apparently healthy adults aged > 18 years. SUA, liver enzymes, lipid profile and other biochemical markers were measured in the collected samples by using standard methods. Multinomial logistic regression model was used to assess the relationship between SUA and elevated levels of liver enzymes among the participants. Overall, the prevalence of hyperuricemia was 30.1% with 32.2% in male and 18.6% in female participants. About 33% of the participants had at least one or more elevated levels of liver enzymes. The mean level of SUA was significantly higher in males (389.3 ± 96.9 µmol/L) than in the female (290.4 ± 89.8 µmol/L) subjects (p < 0.001). There was a significant difference in the mean levels of serum ALT and GGT between the male (34.5 ± 16.0 U/L and 26.7 ± 19.5 U/L, respectively) and female (25.0 ± 13.0 U/L and 19.5 ± 13.2 U/L, respectively) participants (p < 0.001 and p < 0.01, respectively). An increasing trend was observed in the mean levels of serum ALT and GGT across the SUA quartile groups (p < 0.001 and p < 0.01, respectively). SUA showed a positive and significant correlation with serum ALT (p < 0.001) and GGT (p < 0.01). In further statistical analysis after adjustment for potential confounders, SUA showed an independent and significant association with serum ALT and GGT in all regression models. In conclusion, SUA was strongly associated with serum levels of ALT and GGT after adjustment for potential confounders. More prospective studies are needed to clarify the complex relationship between SUA and liver enzymes in the general population.

## Introduction

Uric acid is the final product of purine metabolism and in the human body serum uric acid (SUA) is maintained through its production and excretion process^1,2^. After production, a major portion of the uric acid is excreted in urine or passes through the intestine to regulate its normal levels in the blood. However, increased production and decreased excretion of uric acid from the body leads to a condition known as hyperuricemia. In recent decades, the prevalence of hyperuricemia has increased greatly around the world, with rising trends in both developing and developed countries^2,3^. Hyperuricemia is considered as a cause of gouty arthritis and kidney stones. More recently in epidemiological studies, hyperuricemia has been implicated in the development of metabolic syndrome, hypertension and cardiovascular diseases^4–6^. Moreover, hyperuricemia has been found to be associated with non-alcoholic fatty liver disease (NAFLD)^7–10^.

Liver is a key organ in the human body, responsible for various important functions including synthesis of critical proteins, control of many aspects of metabolism and detoxification of various metabolites^11–13^. The liver also plays a vital role in metabolism, red blood cells regulation and glucose homeostasis^12,13^. Serum enzymes such as alanine and aspartate transaminase (ALT and AST), alkaline phosphatase (ALP), and gamma-glutamyl transferase (GGT) are commonly used in liver functions tests. Serum ALT is considered as a specific marker for hepatic injury and is mainly found in this organ^14–16^, while serum GGT is present in maximum cells surface and highly active in the liver, kidney and pancreas^14^. Serum GGT is regarded as a biomarker of hepatic dysfunctions and alcohol consumption^14^. Furthermore, GGT mediates glutathione uptake and is thought to be connected to oxidative stress and chronic inflammation^14,17,18^. Elevated levels of serum ALT, and GGT are associated with various risk factors for diabetes, metabolic syndrome and cardiovascular diseases such as hyperglycemia, obesity, dyslipidemia and increased blood pressure. Increased levels of ALT and GGT have also been found to be associated with NAFLD^19^.

Since SUA and liver enzymes, especially ALT and GGT, have been associated with various conditions, including increased blood pressure, cardiovascular disorders, NAFLD and metabolic syndrome, these parameters are also likely to demonstrate relationships with each other. In an early study, SUA showed a significant association with the presence of NAFLD in Korean adults^20^. A study in Japan, reported a relationship between serum GGT and SUA in a community-dwelling Japanese inhabitants^21^. Another study showed an association between serum GGT and elevated SUA in normotensive Chinese individuals^22^. A recent study showed a significant positive association and dose-dependent relationship between SUA and the prevalence of NAFLD in nonobese postmenopausal population in China^23^. The elevated SUA concentration, even within the normal range also showed an association with increased ALT in Chinese adults^24^.

There are some reports on the association between SUA and liver enzymes; however, there is still limited information on the direct relationship between SUA level and liver enzyme activity or vice-versa in the general adult population. Although there is some evidence that increased levels of serum GGT influence the development of hyperuricemia, in another way, whether elevated SUA also contributes to the increased levels of liver enzymes in the general population needs further investigations. Therefore, the present study was conducted to assess the potential relationship between SUA and liver enzymes in a Bangladeshi adult population.

## Methods

### Study setting and study population

This cross-sectional study was conducted from December 2018 to November 2019 at the Department of Biochemistry and Molecular Biology, Shahjalal University of Science and Technology, Bangladesh. A total of 410 participants were randomly enrolled from general adults in Sylhet region, university academic and non-academic staff and adult students. Inclusion criteria: (i) both gender aged ≥ 18 years and (ii) participants free from severe illness. Exclusion criteria: (i) age < 18 years, (ii) pregnant women and lactating mother, (iii) subjects with a history of alcohol consumption or drug addiction, (iv) subjects taking anti-diabetic, and antihyperuricemic drugs, (v) subjects with other known chronic hepatic diseases such as viral hepatitis or autoimmune hepatitis, (vi) subjects who had nonalcoholic steatohepatitis (NASH), or drug induced liver injury and (vii) subjects with incomplete data involving blood test values were also excluded to avoid bias. All subjects gave informed consent before their inclusion in the study. This study was approved by the Internal Ethics Review Board at the Department of Biochemistry and Molecular Biology, School of Life Sciences, Shahjalal University of Science and Technology, Bangladesh. All the steps in the method sections were conducted according to the institutional guidelines and regulations.

### Anthropometric data collection

A structured questionnaire was used to collect data on demographic and anthropometric characteristics. Demographic data such as age, sex and anthropometric measurements for example weight, height and waist circumference were administered by trained personnel's using a standardized protocol^6,25–28^. The weight of the participants was measured in kilograms by a digital electronic LCD weighing machine (Beurer 700, Germany) wearing light clothing and no shoes, and height was measured in centimeters using height measuring tape with no shoes and in an upright position. Accordingly, body mass index (BMI) was calculated as weight in kilogram divided by height in meter square (kg/m^2^). Waist circumference (WC) was measured at the midpoint between the lower margin of the last palpable rib and the top of the iliac crest in the mid axillary using a stretch-resistant tape. Blood pressure (BP) was measured digitally using Omron BP M10 (Tokyo, Japan) from the right upper arm.

### Specimen collection and laboratory analysis

About 5 mL of the overnight fasting blood sample was collected from each participant for biochemical analyses. Blood biochemical analyses included serum uric acid (SUA), fasting blood glucose (FBG), triglycerides (TG), total cholesterol (TC), low-density lipoprotein cholesterol (LDL-c), high-density lipoprotein cholesterol (HDL-c), and liver enzymes; alanine aminotransferase (ALT), aspartate aminotransferase (AST), alkaline phosphatase (ALP) and γ-glutamyltransferase (GGT). Serum liver enzyme activities were determined by kinetic methods and other biochemical parameters were measured by standard colorimetric methods. The biochemical parameters were measured by a biochemistry analyzer (Humalyzer 3000, USA) following the manufacturer’s instructions and standard operating procedures.

### Diagnostic criteria

Hyperuricemia was defined as SUA > 7 mg/dL (416.4 µmol/L) in men and SUA > 6 mg/dL (356.9 µmol/L) in women^2^. The elevated level of liver enzymes was defined based on recommended cutoffs as a serum ALT level > 45 U/L for men and > 34 U/L for women, AST level > 35 U/L for men and > 31 U/L for women, GGT level > 55 U/L for men and > 38 U/L for women and ALP level > 128 U/L for men and > 98 U/L for women^29–31^. Participants were identified as having liver dysfunctions or elevated levels of liver enzymes if they had at least one liver enzyme above the upper limit of the reference range.

### Statistical analyses

All data were analyzed by using IBM SPSS version 25. Continuous variables with normal distributions are expressed as the mean ± SD, and categorical variables are presented as percentages (%). Based on the frequency test, SUA was categorized into four quartiles (Q1: ≤ 310 mmol/L, Q2: 311–363 mmol/L, Q3: 364–423 mmol/L and Q4: > 423 mmol/L). Before choosing the parametric tests, the normality of data was assessed by constructing a histogram. Pearson’s correlation was used to determine the correlation between SUA and liver enzymes. The independent sample *t* test (two-tailed) was performed to assess the differences for anthropometric and baseline variables in the gender groups. The differences for baseline variables in the SUA quartile groups were determined by one-way ANOVA. Finally, multiple linear regression and multinomial logistic regression were applied to evaluate the association between SUA levels and liver enzymes among the participants. Potential confounding variables such as age, sex, BMI, FBG, SBP, DBP and lipid profile markers were adjusted in the regression models. Values were considered statistically significant if p < 0.05.

## Results

### Baseline characteristics of the study population

The baseline characteristics of the study population are presented in Table 1 by gender group. A total of 410 participants were included in this study; about 74.1% (n = 304) were males and 25.9% (n = 106) were females. The mean age (± SD) of the study subjects was 36 ± 11 years which range from 20 to 65 years. The mean BMI of the study participants was 24.4 ± 3.5 kg/m^2^ with no significant difference between males and females. The mean level of SBP, SUA, ALT, AST and GGT were significantly higher in males than in the female participants (at least p < 0.05 for all cases). However, the mean level of serum TG and HDL were significantly higher in females than in the male (p < 0.01 for both cases) subjects. There were no significant differences in the mean level of DBP, FBG, TC, and LDL-C, in the male–female groups.

**Table 1:** Baseline characteristics of the study participants according to sex.

	Overall	Male	Female	p-value
Number (n)	410	304	106	–
Age (years)	36.0 ± 11.0	37.0 ± 11.0	34.0 ± 11.0	0.105
Weight (kg)	66.1 ± 11.2	67.6 ± 10.6	58.4 ± 11.5	0.000
Height (cm)	163.8 ± 12.2	166.1 ± 6.4	151.3 ± 23.7	0.000
BMI (kg/m^2^)	24.4 ± 3.5	24.5 ± 3.4	24.3 ± 4.1	0.860
WC (cm)	84.2 ± 12.3	84.9 ± 12.5	80.8 ± 10.6	0.078
SBP (mmHg)	124.0 ± 12.0	125.0 ± 12.0	120.0 ± 13.0	0.041
DBP (mmHg)	83.0 ± 10.0	83.0 ± 10.0	82.0 ± 10.0	0.467
PP (beats/min)	76.0 ± 12.0	75.0 ± 12.0	81.0 ± 12.0	0.006
FBG (mmol/L)	5.2 ± 0.7	5.2 ± 0.7	5.3 ± 0.9	0.355
TG (mg/dL)	172.8 ± 97.3	181.3 ± 100.8	128.4 ± 60.1	0.000
TC (mg/dL)	195.2 ± 59.2	195.2 ± 62.5	195.0 ± 38.6	0.976
HDL-C (mg/dL)	31.7 ± 9.8	31.0 ± 10.0	35.1 ± 7.6	0.004
LDL-C (mg/dL)	130.0 ± 51.9	129.9 ± 54.5	130.5 ± 36.4	0.922
SUA (µmol/L)	374.3 ± 102.1	389.3 ± 96.9	290.4 ± 89.8	0.000
ALT (U/L)	33.1 ± 15.9	34.5 ± 16.0	25.0 ± 13.0	0.000
AST (U/L)	25.1 ± 11.0	25.9 ± 11.1	21.2 ± 10.1	0.036
GGT (U/L)	25.7 ± 18.9	26.7 ± 19.5	19.5 ± 13.2	0.004
ALP (U/L)	97.4 ± 35.5	99.4 ± 35.3	86.7 ± 35.0	0.101
Hyperuricemia (%)	30.1	32.2	18.6	–

Values are presented as mean ± SD. p-values are given for differences between the gender groups, obtained from an independent sample *t* test. *BMI* body mass index, *WC* waist circumference, *SBP* systolic blood pressure, *DBP* diastolic blood pressure, *PP* pulse pressure, *FBG* fasting blood glucose, *TG* triglyceride, *TC* total cholesterol, *HDL-C* high density lipoprotein cholesterol, *LDL-C* low density lipoprotein cholesterol, *SUA* serum uric acid, *ALT* alanine aminotransferase, *AST* aspartate aminotransferase, *GGT* gamma glutamyltransferase, *ALP* alkaline phosphatase.

**Figure 1:**
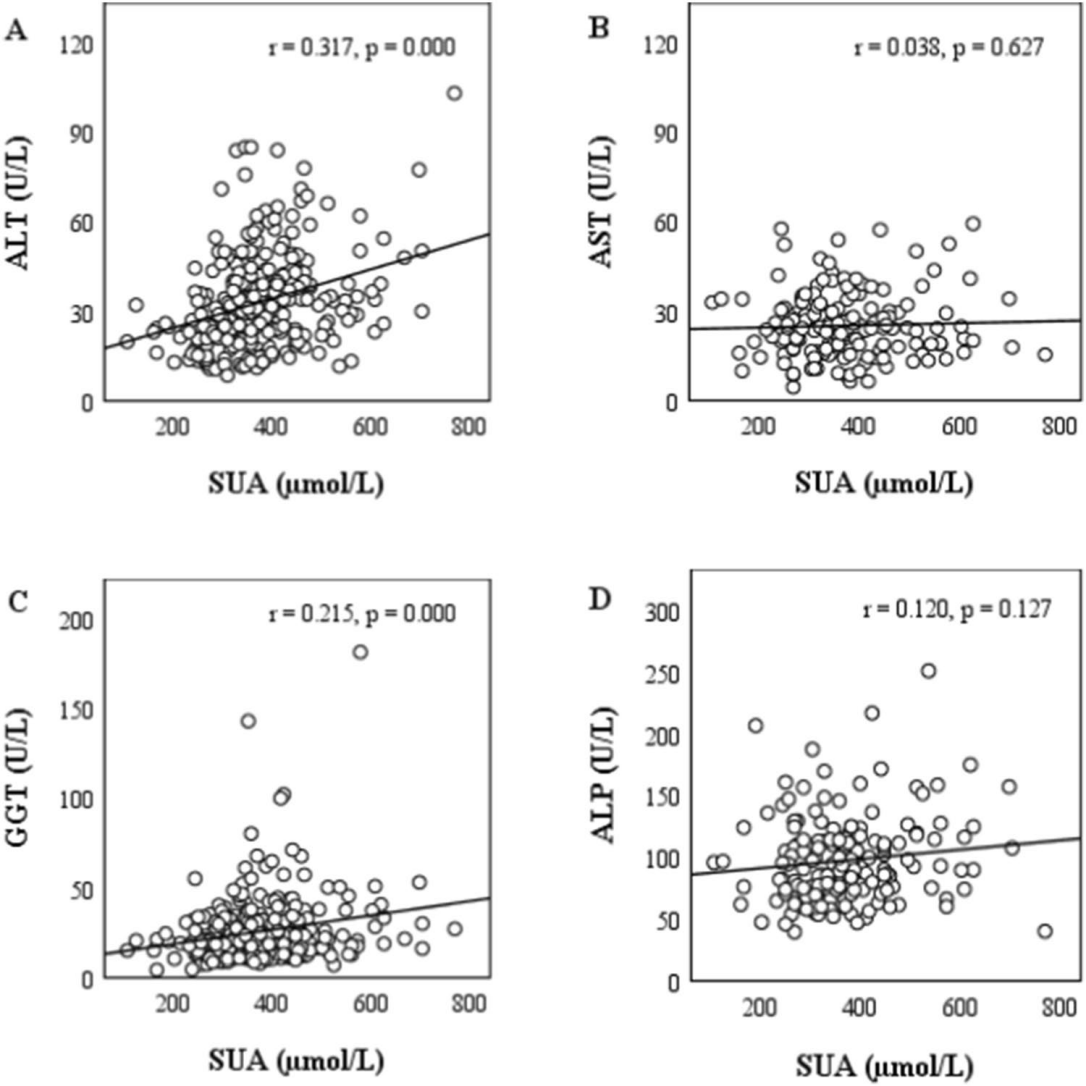
Correlation between SUA and liver enzymes.

The overall prevalence of hyperuricemia was 30.1% with 32.2% in male and 18.6% in female subjects. A significant positive correlation was found between SUA and serum levels of ALT and GGT (p < 0.001 and p < 0.01, respectively) among the participants (Fig. 1). On the other hand, no significant correlation was found between SUA and serum levels of AST and ALP. About 33% of the participants had at least one or more elevated levels of liver enzymes (Table 2). The prevalence of elevated liver enzymes was higher in the fourth SUA quartile group than in other quartile groups.

**Table 2:** Prevalence of elevated liver enzymes across the SUA quartiles.

Liver enzymes	Elevated (%)				
	Overall	Q1	Q2	Q3	Q4
ALT	17.9	8.6	17.6	18.6	27.9
AST	19.4	17.6	19.4	22.2	19.0
GGT	6.2	2.7	7.4	8.7	6.2
ALP	17.2	28.0	11.1	8.3	17.5
One or more elevated enzymes	32.9	32.9	25.0	32.9	41.2

Elevated liver enzymes were defined on the basis of recommended cutoffs as a serum ALT level > 45 U/L for men and > 34 U/L for women, AST level > 35 U/L for men and > 31 U/L for women, GGT level > 55 U/L for men and > 38 U/L for women and ALP level > 128 U/L for men and > 98 U/L for women^29–31^.

### Baseline characteristics of the study population according to SUA quartiles

**Figure 2:**
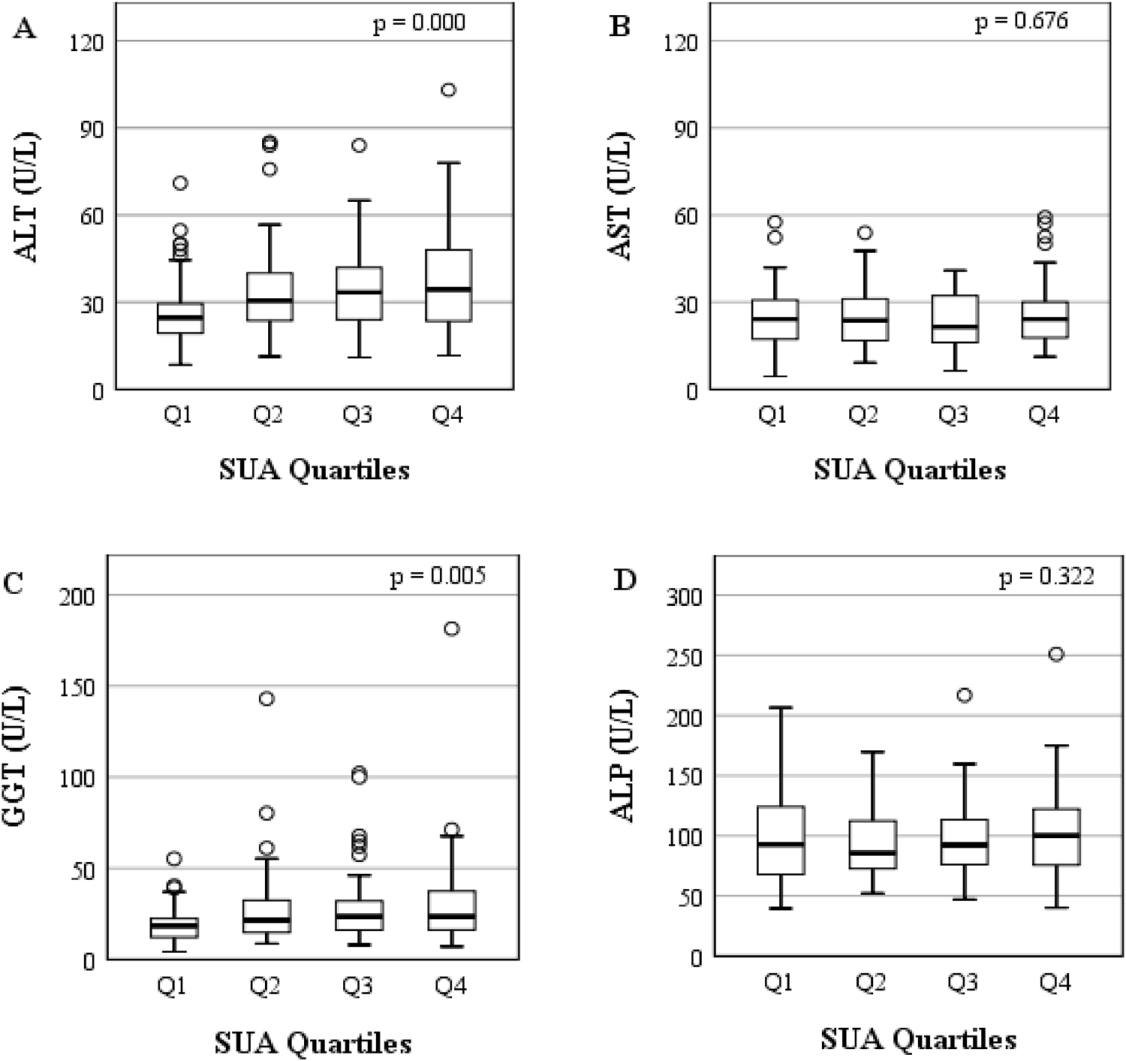
Levels of liver enzymes across the SUA quartiles.

Quartiles according to SUA levels in this study cohort were categorized as follows: Q1: ≤ 310 mmol/L, Q2: 311–363 mmol/L, Q3: 364–423 mmol/L and Q4: > 423 mmol/L. The basic characteristics according to SUA quartiles are summarized in Table 3. There were significant differences in the mean age, BMI, DBP, TG, SUA, ALT, and GGT in the SUA quartile groups (at least p < 0.05 for all cases). An increasing trend of serum ALT and GGT was observed across the SUA quartile groups (p < 0.001 and p < 0.01, respectively) (Table 3 and Fig. 2). There were no significant differences in TC, HDL-C, LDL-C, AST and ALP in the SUA quartile groups.

**Table 3:** Characteristics of the study subjects according to SUA quartiles.

	SUA (µmol/L)				
	Q1 (≤ 310)	Q2 (311–363)	Q3 (364–423)	Q4 (> 423)	p-value
Number (n)	106	102	100	102	–
Age (years)	37.0 ± 11.0	38.0 ± 12.0	34.0 ± 10.0	36.0 ± 12.0	0.208
Weight (kg)	60.6 ± 11.0	65.0 ± 9.5	68.3 ± 10.3	70.7 ± 11.7	0.000
Height (cm)	158.8 ± 20.2	164.4 ± 7.1	166.3 ± 6.7	160.0 ± 6.5	0.000
BMI (kg/m^2^)	23.4 ± 3.4	24.0 ± 2.6	24.7 ± 3.7	25.6 ± 3.7	0.001
WC(cm)	81.3 ± 11.5	82.9 ± 7.8	83.8 ± 11.6	88.1 ± 15.6	0.060
SBP (mmHg)	121.0 ± 12.0	126.0 ± 14.0	125.0 ± 10.0	124.0 ± 12.0	0.064
DBP (mmHg)	80.0 ± 10.0	86.0 ± 12.0	83.0 ± 9.0	81.0 ± 10.0	0.005
PP (beats/min)	76.0 ± 13.0	76.0 ± 10.0	77.0 ± 12.0	76.0 ± 14.0	0.962
FBG (mmol/L)	5.3 ± 0.8	5.1 ± 0.6	5.1 ± 0.7	5.3 ± 0.8	0.052
TG (mg/dL)	144.6 ± 77.5	175.4 ± 101.8	182.0 ± 93.0	193.4 ± 113.8	0.023
TC (mg/dL)	184.1 ± 51.1	203.5 ± 49.6	191.1 ± 54.4	205.2 ± 78.0	0.116
HDL-C (mg/dL)	32.7 ± 9.5	32.6 ± 10.7	29.3 ± 8.4	32.2 ± 10.4	0.142
LDL-C (mg/dL)	121.9 ± 45.5	134.4 ± 45.2	129.6 ± 50.6	136.8 ± 64.6	0.348
SUA (µmol/L)	262.4 ± 42.5	341.7 ± 14.6	393.2 ± 18.0	512.3 ± 78.5	0.000
ALT (U/L)	25.8 ± 11.3	33.8 ± 16.5	35.0 ± 14.7	38.3 ± 18.0	0.000
AST (U/L)	24.7 ± 10.5	26.0 ± 11.0	23.5 ± 10.4	26.2 ± 12.3	0.676
GGT (U/L)	19.2 ± 9.7	26.8 ± 19.9	27.6 ± 18.4	29.7 ± 23.9	0.005
ALP (U/L)	95.9 ± 37.2	92.1 ± 28.1	95.9 ± 33.0	106.3 ± 40.4	0.322

Values are presented as mean ± SD. p-values were obtained from one-way ANOVA. *BMI* body mass index, *WC* waist circumference, *SBP* systolic blood pressure, *DBP* diastolic blood pressure, *PP* pulse pressure, *FBG* fasting blood glucose, *TG* triglyceride, *TC* total cholesterol, *HDL-C* high density lipoprotein cholesterol, *LDL-C* low density lipoprotein cholesterol, *SUA* serum uric acid, *ALT* alanine aminotransferase, *AST* aspartate aminotransferase, *GGT* gamma glutamyltransferase, *ALP* alkaline phosphatase.

### Association between SUA and liver enzymes

In further statistical analysis, we applied both multiple linear regressions and multinomial logistic regression to evaluate the relationship between SUA and liver enzymes. Some confounding variables such as age, sex, BMI, FBG, SBP, DBP and lipid profile markers were adjusted in the regression models. In both regression analyses, a positive and independent association was observed between SUA and serum ALT and GGT among the participants (Tables 4, 5).

**Table 4:** Multiple linear regressions to assess the relationship between SUA and liver enzymes.

	ALT			AST			GGT			ALP		
	B	95% CI	p-value	B	95% CI	p-value	B	95% CI	p-value	B	95% CI	p-value
Model 1	0.050	0.030–0.070	0.000	0.006	− 0.008 to 0.021	0.402	0.042	0.019–0.066	0.000	0.044	− 0.004 to 0.092	0.072
Model 2	0.044	0.023–0.065	0.000	0.007	− 0.008 to 0.022	0.370	0.031	0.007–0.055	0.011	0.046	− 0.006 to 0.098	0.082
Model 3	0.041	0.019–0.062	0.000	0.005	− 0.010 to 0.021	0.515	0.031	0.006–0.056	0.016	0.042	− 0.012 to 0.095	0.126

Multiple linear regression analysis was done to assess the relationship between SUA and liver enzymes. Dependent variable is liver enzymes (U/L) and the independent variable is SUA (µmol/L). Model 1: adjusted for age (years). Model 2: model 1 + BMI and FBG. Model 3: model 2 + SBP, DBP and lipid profile markers.

**Table 5:** Association between SUA and liver enzyme across the quartiles.

SUA level (µmol/L)					
	Q1 (≤ 310)	Q2 (311–363)	Q3 (364–423)	Q4 (> 423)	p for trend
**ALT**					
Model 1	1.00 (Ref.)	1.050 (1.021–1.080)	1.048 (1.018–1.079)	1.057 (1.028–1.087)	0.000
Model 2	1.00 (Ref.)	1.044 (1.015–1.074)	1.039 (1.009–1.070)	1.049 (1.019–1.079)	0.002
Model 3	1.00 (Ref.)	1.033 (1.003–1.063)	1.031 (1.000–1.063)	1.039 (1.009–1.070)	0.027
**AST**					
Model 1	1.00 (Ref.)	1.007 (0.966–1.050)	0.979 (0.933–1.026)	1.005 (0.966–1.047)	0.619
Model 2	1.00 (Ref.)	1.019 (0.974–1.065)	0.976 (0.927–1.028)	1.007 (0.962–1.054)	0.390
Model 3	1.00 (Ref.)	1.010 (0.958–1.065)	0.961 (0.905–1.020)	1.009 (0.960–1.061)	0.277
**GGT**					
Model 1	1.00 (Ref.)	1.055 (1.022–1.089)	1.055 (1.022–1.090)	1.058 (1.025–1.093)	0.000
Model 2	1.00 (Ref.)	1.052 (1.018–1.087)	1.048 (1.013–1.084)	1.047 (1.013–1.083)	0.003
Model 3	1.00 (Ref.)	1.049 (1.013–1.087)	1.047 (1.010–1.085)	1.045 (1.008–1.083)	0.009
**ALP**					
Model 1	1.00 (Ref.)	0.995 (0.981–1.009)	0.999 (0.984–1.013)	1.006 (0.994–1.018)	0.419
Model 2	1.00 (Ref.)	0.996 (0.983–1.010)	1.000 (0.985–1.015)	1.008 (0.995–1.021)	0.427
Model 3	1.00 (Ref.)	0.996 (0.979–1.012)	0.995 (0.978–1.013)	1.013 (0.998–1.028)	0.119

Multinomial logistic regression analysis was done to assess the relationship between SUA and liver enzymes. Dependent variable is SUA (µmol/L) and the independent variable is liver enzymes (U/L). Reference category is SUA quartile Q1. Model 1: adjusted for age (years) and sex (male and female). Model 2: model 1 + BMI and FBG. Model 3: model 2 + SBP, DBP and lipid profile markers. *OR* odds ratio, *CI* confidence interval.

## Discussion

In the present study, SUA was positively associated with serum ALT and GGT, independent of confounding factors such as age, sex, BMI, FBG, SBP, DBP and lipid profile markers. Furthermore, we observed that SUA was not only related to serum liver enzymes but also an independent predictor of the increased ALT and GGT.

The possible role of SUA in NAFLD has been investigated in some epidemiological studies^8,9,32^. However, little is known about the direct relationship between SUA and liver enzymes in the general adult population. Therefore, we performed this cross-sectional study to evaluate the association between SUA and the maximum number of liver enzymes in a Bangladeshi adult population. The overall prevalence of hyperuricemia and liver enzymes abnormalities were about 30% and 33%, respectively, among the participants. The prevalence of hyperuricemia and liver enzymes abnormality were comparatively higher in male than in female participants. We also observed that serum levels of ALT and GGT were gradually increased according to the SUA concentrations across the quartile groups. In regression analysis, SUA showed a strong and independent association with serum ALT and GGT after controlling potential variables.

Our results are in line with the findings of a few previous studies that demonstrated a positive relationship between SUA and liver enzymes, especially GGT or vice versa. For example, a study by Zhang et al. reported a positive association between serum GGT and increased SUA levels in normotensive Chinese adults free of kidney, liver and metabolic disease^22^. Another study showed a positive relationship between serum GGT and SUA in a community-dwelling Japanese population, in which the hypertensive participants were included^21^. A hospital-based study in Spain reported that hyperuricemia in the context of alcohol use disorder was associated with serum GGT and suggesting an increased cardio-metabolic risk in the study population^33^. Further study in Taiwan indicated hyperuricemia as an independent risk factor that was associated with elevated levels of serum ALT in obese adolescents^34^. In a nationally representative survey that included 14,407 participants in the USA, the level of SUA was associated with the incidence of cirrhosis and elevated serum ALT and GGT^7^. One of the major limitations of that study was that diagnosis of cirrhosis was confirmed based on hospitalization data and death certificates^7^. The authors also suggested that future studies should be conducted to investigate whether the association between SUA and liver enzymes is causal or just a marker of NAFLD or influence the progression of alcoholic or viral hepatitis^7^.

A possible link between SUA and the incidence of NAFLD has been suggested in previous studies. Such as, a prospective study in China showed elevated SUA as an independent predictor for incident NAFLD^8^. In a study in the USA, NAFLD was found to be higher among participants who had higher levels of SUA^9^. A 5-year retrospective study indicated higher SUA as a possible risk factor for the development of NAFLD in apparently healthy adults in Korea^32^. These studies suggest a relationship between hyperuricemia and NAFLD; this would be expected as hyperuricemia is associated with various risk factors for NAFLD, such as metabolic syndrome, insulin resistance and obesity^7^.

In the present study, SUA showed an association with serum liver enzymes levels especially ALT and GGT. However, the mechanism underlying is not well understood yet, but a possible explanation for it might be insulin resistance. It has been suggested that insulin resistance may cause a reduction of urate excretion via the kidney, and this reduction, in turn, may increase SUA levels in blood^35,36^. Furthermore, insulin resistance may promote purine metabolism through activation of hexose monophosphate Shunt pathway^37^.

It has been reported that hyperuricemia, rather than just a simple marker, might contribute to the cause of oxidative stress, insulin resistance, metabolic syndrome and systemic inflammation^4,38^. Because these conditions are associated with NAFLD and can lead to steatohepatitis or even enhance the progression of alcoholic or viral hepatitis^7^. It has been proposed that hyperuricemia-induced endothelial dysfunction may be a potential reason that promotes insulin resistance by impairing nitric oxide release. In animal studies, hyperuricemia has been shown to be associated with oxidative and inflammatory changes in adipocytes which are responsible for developing metabolic syndrome in obese mice^39^. Yet, it remains unclear whether hyperuricemia is the cause or result of conditions that influence liver disease progression is of considerable interest as pharmacological agents are effective in reducing SUA levels only if hyperuricemia is a cause rather than a result of these conditions. Similarly, hyperuricemia is a cause or effects of cardiovascular disease are still under investigations^4,38^. Nonetheless, even if hyperuricemia is proved only a marker and not a cause of hepatic necroinflammation and cirrhosis in future studies, it is still an effective marker as it can predict the liver enzymes abnormalities independently of other available predictors currently used. Further studies are needed to elucidate the exact mechanism involved in such a relationship.

There were some limitations in the present study. Firstly, this study was cross-sectional design; therefore, it is not clear whether SUA has a potentially causal role in elevating liver enzymes in general adults. Secondly, the number of participants enrolled in the study was relatively small; therefore, our findings may not represent the entire population in Bangladesh. Thirdly, the study only includes Bangladeshi adults, suggesting that our study findings should be confirmed in other ethnicities. Although further studies are needed, our study findings indicate that SUA may be a useful predictor of liver enzymes abnormalities in the general population.

## Conclusions

The present study showed a strong and positive association between SUA and serum ALT and GGT in Bangladeshi adults, independent of confounding factors. Our findings support the fact that increased levels of SUA may be an effective marker in predicting liver enzymes abnormalities especially for serum ALT and GGT. However, prospective studies are needed to understand the underlying mechanisms involved in the relationship between SUA and liver enzymes.
